# A Group of London Medical Schools

**Published:** 1916-12-09

**Authors:** 


### FURTHER PATRIOTISM IN BRITISH HOSPITALS.

### A Group of London Medical Schools.

### St. Thomas's Hospital.

The following brief synopsis of the services rendered to the nation by the past and present students of St. Thomas's Hospital speaks for itself:—

Joined the Navy and Army, 655; serving in the Regular Forces before the war, 128; total, 783; died in the service of their country, 228; K.C.M.G., 1; C.B., 1; M.V.O., 1; C.M.G., 7; D.S.O., 8; the Military Cross, 9; Victoria Cross (Captain G. A. Maling), 1; Mentioned in Dispatches, 58.

Captain L. G. Bourdillon was awarded the Military Cross and D.S.O.

Serbian, Belgian, and Egyptian Honours, 8.

### St. George's Hospital.

A very large number of the past and present students of St. George's Medical School are doing work for the wounded. Thirty-eight are serving in the Navy and 143 in the Army.

The number of the staff of the hospital spared for active war service is ten; nearly all of the remainder hold commissions in the R.A.M.C. Territorial Force, and are on duty from time to time at the 4th London General Hospital.

Among the honours earned by members of the staff and students are: 1 C.B., 4 C.M.G., 1 C.I.E., 2 D.S.O., 3 Military Cross, 17 Mentioned in Dispatches.

The special war work undertaken by the hospital is the allocation of 100 beds for the treatment of the wounded, and the bacteriological laboratory staff have undertaken the inoculation against typhoid and allied fevers of motor-ambulance men of the British Red Cross Society, the Service de Santé Militaire, and members of the Croix Rouge Français.

### London School of Clinical Medicine: Seamen's Hospital, Greenwich.

The following members of the staff have joined H.M. Forces since the outbreak of the war as members of the Royal Army Medical Corps or the Indian Medical Service:—

Sir John Rose Bradford, K.C.M.G., C.B., F.R.S.; Messrs. E. Rock Carling, F.R.C.S.; R. E. Bickerton, M.B. (Mentioned in Dispatches); C. C. Choyce, F.R.C.S. (Mentioned in Dispatches); G. N. Biggs, M.B., B.S.; H. McCormac, M.R.C.P.; Cecil Rowntree, F.R.C.S.; Colonel T. D. Collis Barry; C. E. Sundel, M.D.; Gordon Holmes, M.D.; H. B. Carlill, M.D.; R. S. Lawson, M.B., Ch.B.; Arthur Davies, M.D. Nine students are also doing military service in the R.A.M.C., the I.M.S., or in the ranks.

Lieutenant B. C. Pring, the almoner, and Mr. Maxted Hughes, clerk in the accountant's department, have been killed in action.

Being a post-graduate school, the authorities of the Seamen's Hospital have not been able to keep in such close touch with their students as is done at other schools of medicine. It is probable, therefore, that the above brief account by no means represents the extent to which the teaching of the hospital has borne fruit in devotion to the wounded.

